# Close to Transplant Renal Artery Stenosis and Percutaneous Transluminal Treatment

**DOI:** 10.1155/2011/219109

**Published:** 2011-07-05

**Authors:** Leonardou Polytimi, Gioldasi Sofia, Pappas Paris

**Affiliations:** Department of Radiology, Laikon General Hospital of Athens, 17 Ag. Thoma street, 115 27 Athens, Greece

## Abstract

*Purpose*. To evaluate the efficacy of percutaneous transluminal angioplasty (PTA) in the management of arterial stenosis located close to the allograft anastomosis (close-TRAS). *Materials and Methods*. 31 patients with renal transplants were admitted to our institution because of persistent hypertension and impairment of transplant renal function and underwent angiography for vascular investigation. 27 were diagnosed suffering from transplant renal artery stenosis (TRAS), whereas 4 had severe iliac artery stenosis proximal to the transplant anastomosis (Prox-TRAS). 3 cases of TRAS coexisted with segmental renal arterial stenosis, whereas 3 other cases of TRAS were caused by kinking and focal stenosis in the middle of the transplanted renal artery. *Results*. Angioplasty and stenting were successfully applied to all patients with iliac artery stenosis as well as to those with TRAS and segmental artery stenosis. Two of three patients with kinking were well treated with angioplasty and stenting, whereas one treated only with angioplasty necessitated surgery. No major procedure-related complications appeared, and the result was decrease of the serum creatinine level and of the blood pressure. *Conclusions*. PTA is the appropriate initial treatment of TRAS and close-TRAS, with low morbidity and mortality rates, achieving improvement of graft function and amelioration of hypertension.

## 1. Introduction

Hypertension is a recognized complication following renal transplantation, occurring in approximately 60% of the recipients, and it is a major risk factor for accelerated cardiovascular disease and mortality after transplantation [1, 2]. The etiology of posttransplant hypertension is multifactorial including many conditions such as acute and chronic rejection, immunosuppressant therapy, graft dysfunction, and renal artery stenosis [3].

TRAS is a well known cause of posttransplant hypertension, and its incidence varies from 1% to 23% depending on the study and the transplantation center, reflecting the heterogeneous criteria used to establish the diagnosis and the presence of asymptomatic lesions [4–6]. Arterial complications lead to graft dysfunction and loss in approximately 5% of all renal transplant recipients [7, 8]. 

The most common arterial complication following kidney transplantation is TRAS, presented with resistant hypertension and serum creatinine elevation.

Our special issue on this abstract, concerning patients with TRAS, is the so-called close-TRAS, id stenosis of proximal iliac or segmental renal arteries and the transplant renal artery stenosis plus kinking. 

Herein we report 3 patients among 31 with clinical manifestations of TRAS who were diagnosed with TRAS and segmental arterial stenosis of graft as well; after undergoing angiography, both of TRAS and segmental arterial stenosis were managed with PTA without any failure.

Although Prox-TRAS is rare, it should be considered in patients with renal dysfunction and resistant hypertension since it has similar clinical symptoms to those of TRAS [9, 10]. Therefore, angiographic examination of patients with hypertension after transplantation should include evaluation of the transplanted renal artery as well as of the aortoiliac axes. This paper presents also four patients who developed arterial hypertension because of proximal iliac artery stenosis.

According to the literature, many factors can provoke TRAS such as chronic rejection, kinking, or compression of a long renal artery and atherosclerotic disease [11]. In this paper, we present also 3 cases of TRAS caused by kinking and focal stenosis in the middle of renal transplanted artery.

## 2. Materials and Method

In a series of 627 renal transplants, performed in our service between January 1999 until June 2010 in a total of 27 patients (15 men and 12 women, aged between 26 to 66 years old (mean 47 years)), 12/27 from cadaveric and 15/27 from living donors presented with refractory hypertension and increased creatinine blood level. In particular, in all 27 patients, systolic and diastolic blood pressure and the number of antihypertensive drugs at the time of diagnosis of the stenosis had significantly increased compared to the time period before or early after renal transplantation (RTX). All patients showed impairment of renal function (as documented by eGFR). With regard to vascular risk factors hypertension was present in 27/27 patients, diabetes mellitus in 12/27, hyperlipidemia in 17/27, smoking in 8/27, hyperuricemia in 22/27, and dialysis duration ranged between 1 to 12 years. Seven patients had preexisting renovascular disease. The time between transplantation and deterioration of renal function or hypertension ranged between 2 days and 89 months (mean: 14 months). In 14 of 27 patients this time was less than 6 months (mean: 3.7 months). Color Doppler Ultrasound (CDU) proceeded the angiographic investigation, based on the following diagnostic protocol. After measurement of peak-systolic (*V*_max_) and end-diastolic (*V*_min_) velocities within interlobar arteries of the upper pole, midportion, and lower pole of the kidney, *V*_mean_ was calculated from the area under the curve of one pulse cycle. The Doppler waveform was analyzed, and the Persistence Index (PI) was calculated according to the formula

(1) $$\text{PI}=\frac{\left(V_{\max\;}-V_{\min\;}\right)}{V_{\text{mean}}}.$$

At least three PIs were averaged over time to give a mean value. Further, peak-systolic velocities within the graft arteries were measured under continuous correction of the Doppler beam angle.

TRAS was suspected in case of an increase of  *V*_max_ > 150% within the stenosis as compared to the pre- or poststenotic flow velocity. Further, intrarenal pulsus parvus et tardus was used as an indirect sign for stenosis. Prox-TRAS was assumed in case of a high systolic peak velocity (*V*_max_ > 200 cm/s) within the aortoiliac region and if the triphasic form of the flow curve was lost distal to the anastomosis with the transplant artery. Pulsus parvus et tardus and/or small PI values (<1.0) without presence of TRAS were also indirect signs for Prox-TRAS.

**Figure 1:**
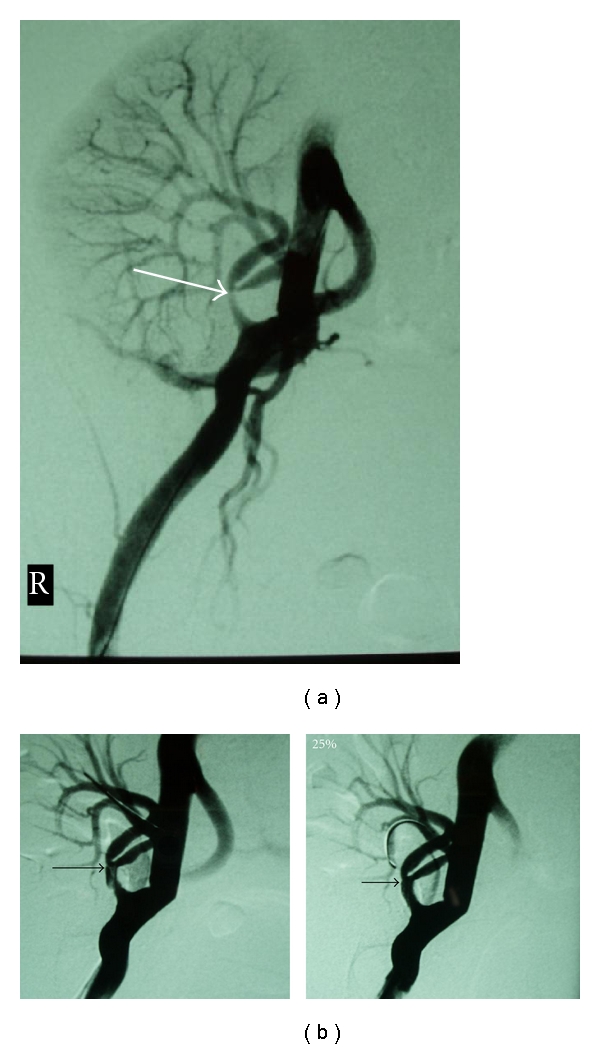
(a) Transplant renal artery stenosis at the bifurcation, affecting mostly the inner ramus (arrow). (b) *Left.* Postballoon angioplasty; dilatation of the segmental renal artery with dissection of the main renal artery lumen (arrow). *Right.* Image Postballoon expandable stent placement (arrow).

**Figure 2:**
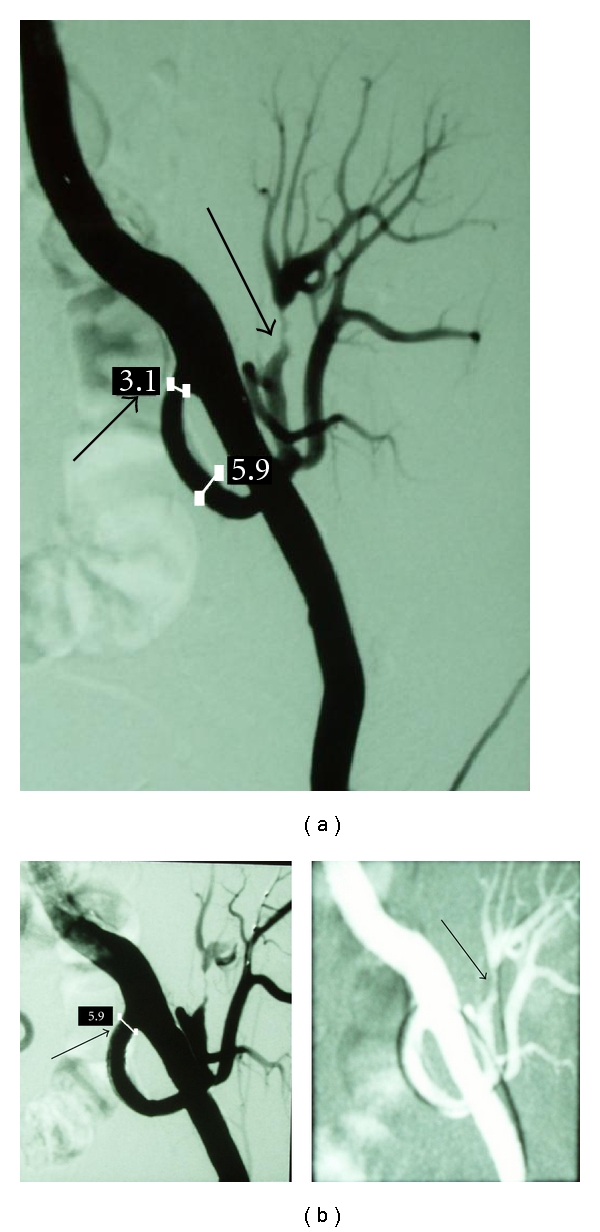
(a) Angiography of the iliac artery revealing the anastomotic stenosis of the transplant renal artery (small arrow) as well as stenosis of the upper segmental renal artery (big arrow). (b) *Left.* Postangioplasty with stent placement at the proximal transplant renal artery (arrow). *Right.* After slight dilatation of segmental renal artery with a thin coronary balloon (arrow).

Among these 27 patients experiencing TRAS, three were identified to suffer also from segmental arterial stenosis (incidence rate 11% of recipient population undergone TRAS (Figures 1(a) and 2(a)).

These three patients presented with persistent hypertension (161 ± 12 and 86 ± 7 mmHg), renal dysfunction (eGFR calculated with Cockroft-Gault formula 22.1 ± 11.4 ml/min), and low PI (<1.0). Diagnostic angiography followed which revealed significant stenosis of partial branch of renal artery apart from the stenosis of the main renal artery. Segmental lesion was dilated by use of a thin, cardiologic balloon of 2.5 mm of diameter, whereas TRAS was managed with stent implantation apart from angioplasty (Figures 1(b) and 2(b)).

We also refer to 4 patients who developed persistent arterial hypertension (168 ± 11 and 84 ± 7 mmHg) despite the increase of prescribed antihypertensive medication and impairment of renal function (eGFR 25 ± 8 ml/min) raising high suspicion of TRAS. CDU investigation illustrated the presence of pulsus parvus et tardus in transplant renal and intrarenal arteries, and PIs were slightly below the normal range (0.9 ± 0.3). The interval between transplantation and graft dysfunction ranged from 2 months up to 4 years. 

**Figure 3:**
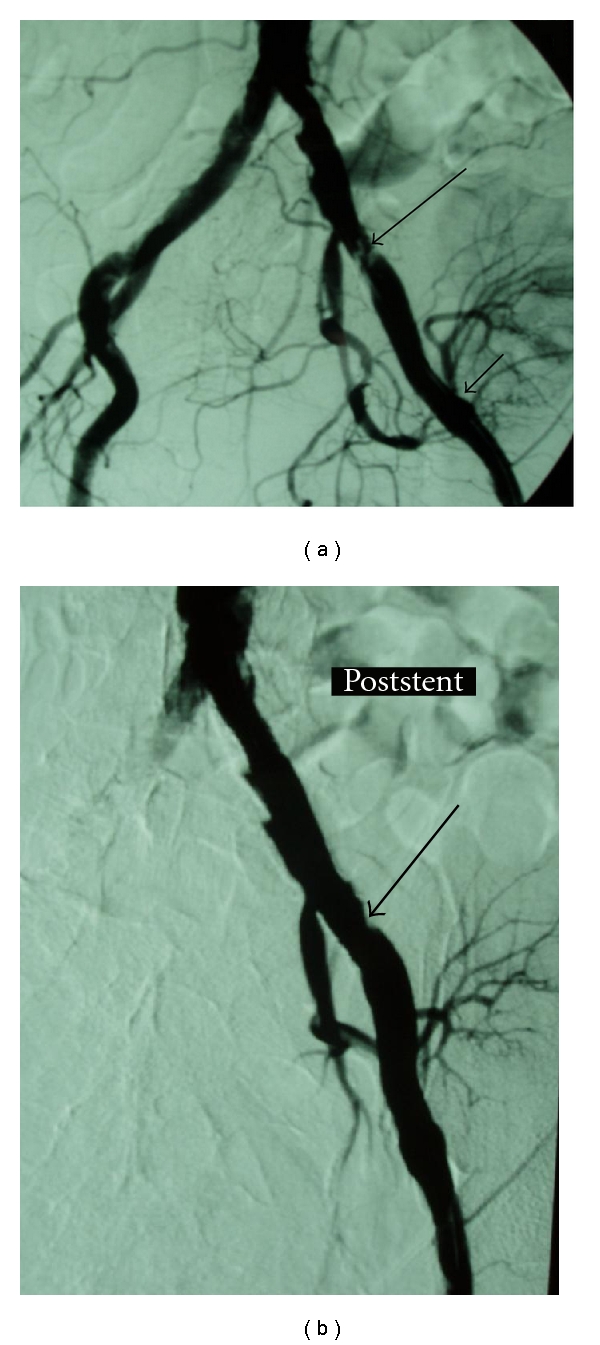
(a) Atheromatous lesions in the iliac arteries, especially at the proximal part of the left external iliac artery (big arrow). Note the anastomosis of the transplant renal artery to the left external iliac artery, a few centimeters distal to this lesion (small arrow). This severe stenosis of the left external iliac artery was responsible for claudication as well as graft's dysfunction. (b) Image post-angioplasty and stent deployment in the left iliac artery (arrow).

**Figure 4:**
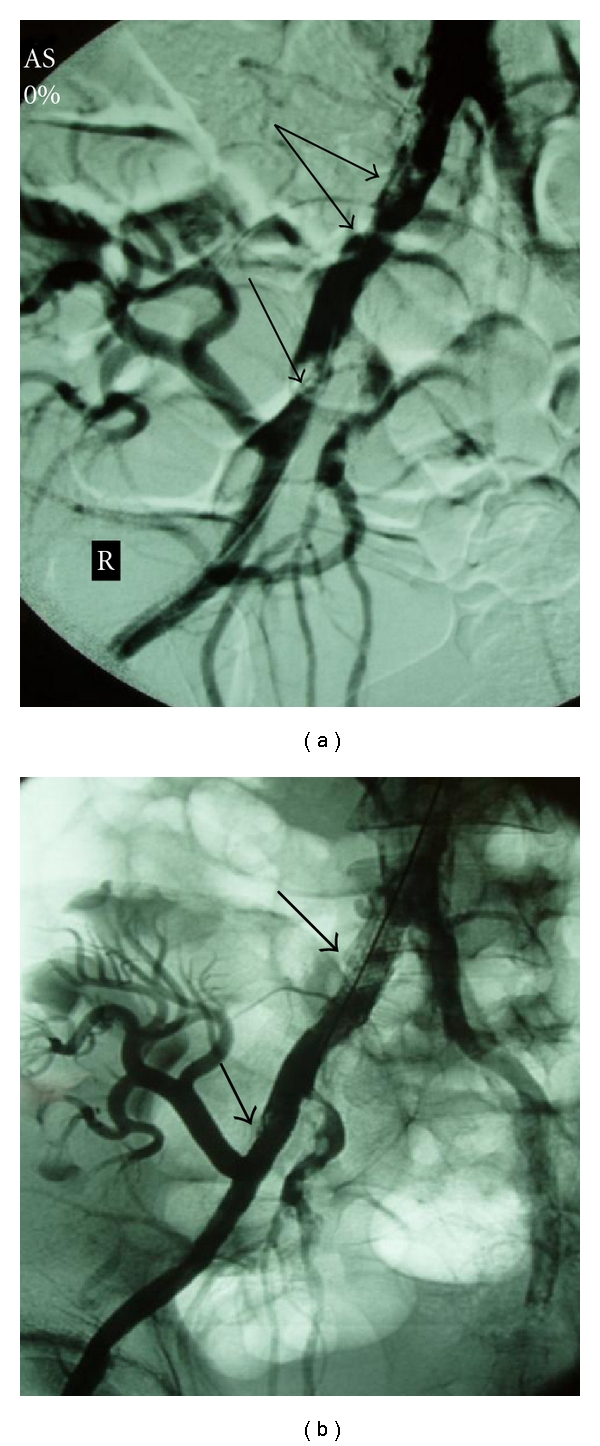
(a) Common and external iliac artery stenosis (arrows) provoking claudication and renal insufficiency. (b) Image following angioplasty and stenting of the common and external iliac artery stenosis (arrows).

Angiography revealed severe ipsilateral iliac artery stenosis (>70%), proximal to the anastomotic site (Figures 3(a) and 4(a)). The cause of Prox-TRAS was atherosclerosis reflecting the disease of the recipients, as these were characterized by coronary artery occlusive disease, older age, and longer time on dialysis before RTX. Successful angioplasty and stenting were performed in all four patients by insertion of an 8 mm diameter, 40 mm length balloon expandable stent, immediately after the angiography in most of the cases (Figures 3(b) and 4(b)).

**Figure 5:**
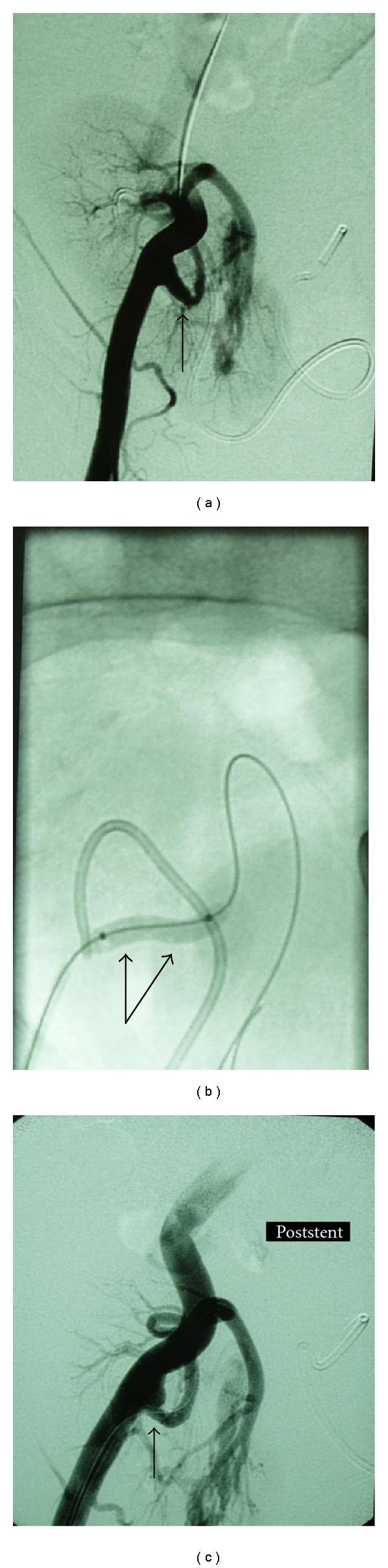
(a) Contralateral angiography revealing kinking with stenosis of the transplant renal artery (arrow). (There is a working tilting of the machine. Note also the ureteral endoprosthesis.) (b) Balloon dilatation of the renal artery stem (arrows) did not succeed to reform the vessel. (c) After stent placement the kinking and stenosis almost disappeared (arrow).

**Figure 6:**
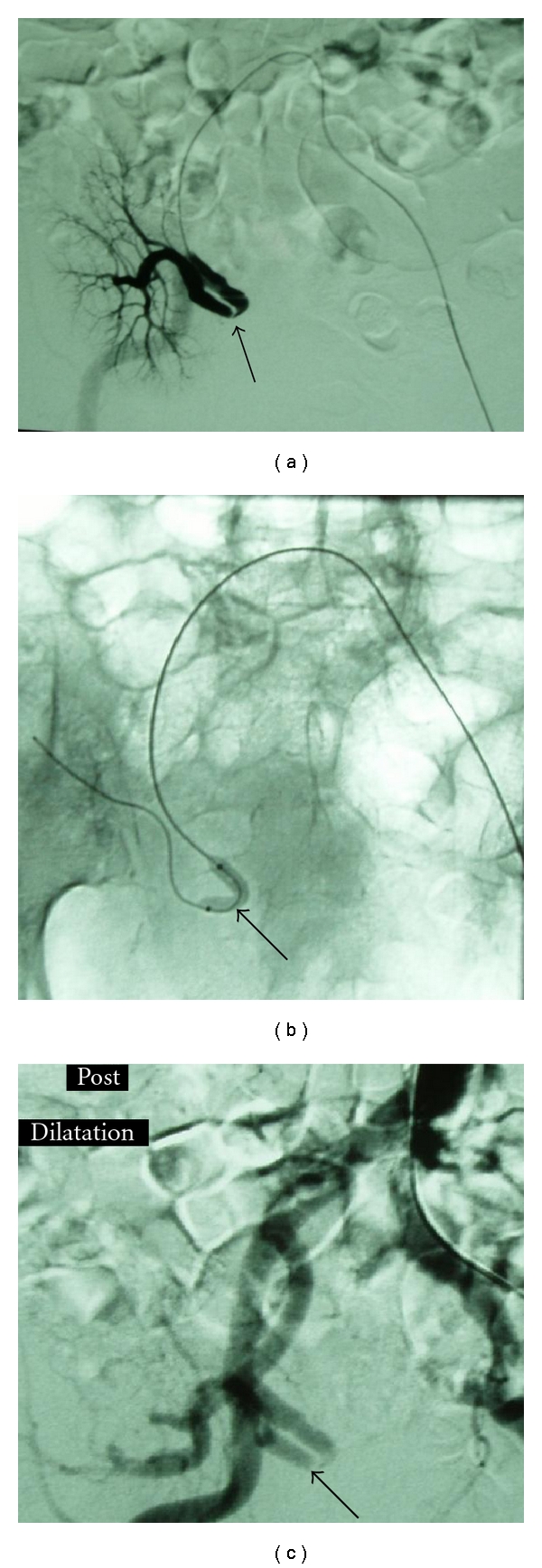
(a) Contralateral selective catheterization of the transplant renal artery shows the kinking and stenosis (arrow). This patient underwent the renal transplantation before 15 days. (b) Balloon angioplasty of the renal artery (arrow). Stent placement was not successful on this particular patient. (c) Angiography after balloon dilatation. The artery does not appear any improvement (arrow).

A subgroup of 3 recipients was identified, in which TRAS resulted from kinking of the renal artery and from segmental stenosis in its medial part (Figures 5(a) and 6(a)). The documented manifestations were hypertension (165 ± 14 and 87 ± 8 mmHg), despite the increase in antihypertensive medication from 2 to 3, deterioration of renal function (eGFR 26 ± 9 ml/min), and low PI values (0.8 ± 0.1) in CDU investigation. The first attempt was to resolve the obstructed lesion in all three patients by means of angioplasty (Figures 5(b) and 6(b)), without any positive outcome in imaging evaluation (Figure 6(c)), therefore, the insertion of a stent followed, which was successfully performed in 2 patients (Figure 5(c)), while the remained one underwent open surgery. 

## 3. Results

### 3.1. Segmental

Resolution of both stenotic sites was achieved in all three patients with TRAS and segmental renal artery stenosis, and no major related-to-the procedure complication took place.

### 3.2. Prox-TRAS

This method was technically feasible in all 4 patients with Prox-TRAS (100%), without any procedure-related complication.

### 3.3. Kinking

PTA with stenting was successfully performed in 2 of 3 cases with kinking (66%)—or otherwise 30/31 id 96.7%. Open surgery was performed to one patient without any major complication related to the procedure, and there was immediate response to treatment in all 3 cases.

During the first 7 postoperative days nearly all patients showed normalization of renal function and decline of arterial blood pressure. In particular, in the subgroup of patients with Prox-TRAS diastolic and systolic blood pressure was reduced to 128 ± 5.6/81 ± 3 mmHg one week after intervention and 122 ± 6.3/74 ± 3 mmHg at the end of observation, number of antihypertensive drugs was declined to 2 and 1 at the mentioned time points, and eGFR was 35.9 ± 11 ml/min post-PTA and 38.9 ± 12.3 at the end of observation. Besides in the subgroup of patients with TRAS and segmental artery stenosis the recorded parameters were blood pressure 129 ± 4/79 ± 3 mmHg, eGFR 37.4 ± 10.4 ml/min one year after PTA and 131 ± 3/79 ± 2 mmHg, eGFR 40.4 ± 12.5 at the end of observation. There was also reduction in the number of antihypertensive medication from 3 to 1. Last at the previously mentioned time points, in the subgroup of patients with TRAS and kinking, we documented systolic and diastolic pressure of 131 ± 6/80 ± 5 mmHg and 130 ± 5/79 ± 4 mmHg, decreased number of antihypertensive drugs from 3 to 2 and to eGFR 31 ± 11 min/mL and 38 ± 9 min/mL, respectively.

Apart from what is mentioned above, the main group of patients with anastomotic TRAS had a successful angioplasty and stenting with a clear amelioration of renal function and hypertension parameters.

In addition, after TRAS was corrected, the ultrasonographic procedure was repeated at 3, 6, and 12 months and yearly thereafter. We determined the peak systolic velocity (*V*_max_), the end-diastolic velocity (*V*_min_), and mean velocity (*V*_mean_) in order to calculate PI, using the same formula mentioned before. The PI values were the average of three measurements in segmental arteries from the upper, middle, and lower third of the kidney. PI increased significantly from 1.1 ± 0.2 to 1.8 ± 0.3 after dilatation of stenosis while with respect to Prox-TRAS the flow profile within the distal segment of the iliac artery became triphasic, whereas it was still monophasic or revealed positive end-diastolic flow proximal to the anastomosis. At the end of observation time PI had slightly decreased from 1.8 ± 0.3 to 1.5 ± 0.2.

During the follow-up period no recurrence had been recorded. Two of the 27 patients died from irrelevant causes (one from heart attack and one from sepsis) with a normally functioning renal transplant. The remaining 25 patients have stable renal function, thus our patency rate, including the operated graft, reached 100%.

## 4. Discussion

### 4.1. Segmental

As evidenced by review of the literature, there is lack of reports concerning segmental artery stenosis of the transplanted kidney, alone and it is challenging to identify its participation in graft's dysfunction. In our opinion, if segmental artery stenosis happened alone, early, significant clinical manifestations would not be present, and therefore, no treatment option would have been employed, since it was a random finding while investigating TRAS of the transplanted kidney with angiographic means.

In addition, PTA is the goldstandard procedure for the management of segmental TRAS since its reported success rate ranged from 70% to 90%, with low complication and recurrence rate [4, 12].

Although this study is limited by its small group of patients, it is suggestive that PTA with or without stenting can be as effective and safe dealing with two stenotic sites as dealing with TRAS alone.

### 4.2. Prox-TRAS

TRAS can be provoked by multiple reasons, depending on its location and the mean time lapse between transplantation and its onset. In particular, stenosis occurring later after transplantation usually reveals atherosclerotic disease either of the renal transplant artery or of the proximal iliac artery [13]. Prox-TRAS is rare, but its clinical manifestations mimic those of TRAS when it appears late after transplantation in patients with stenosis presenting immediately after transplantation, whereas in patients who already have established stable renal function, apart from renovascular hypertension and ischemic nephropathy, there are also signs of peripheral arterial obstruction disease of the lower extremity since peripheral artery resistance has already been developed [7, 9]. As discussed by Humar et al. [14], physical examination of a renal recipient who suffered from resistant hypertension and graft dysfunction revealed a diminished femoral pulse and a significantly lower ankle-brachial index on the right (versus left) lower extremity, that suggested a stenosis of right iliac artery which was afterwards confirmed by angiographic investigation.

As older persons are accepted as transplantation candidates, and as recipient population ages, the presence of occlusive atherosclerosis of the native aortoiliac system or its development proximal to the graft should be expected [15]. Therefore follow-up examination should take into account vascular problems that are related to renal transplantation, as early diagnosis of them leads to sufficient management of graft's impairment. Colour Doppler Ultrasonography and Magnetic Resonance Angiography (MRA) may arouse suspicion of disease even in less symptomatic cases; however definite diagnosis of significant arterial stenosis is accomplished by arteriography combined with angioplasty and stenting when indicated [11, 16]. Moreover in our center the established practice is to look into clinical manifestations of allograft's malfunction and severe hypertension by angiography procedure regardless of the findings of tomographic means [9].

In this paper, the treatment of iliac artery stenosis proximal to renal anastomosis was successful 100% and remained patent during at least 3 years of followup in all four patients. As discussed in other studies, technical success rate of PTA in treatment of Prox-TRAS is recorded to reach 95% while its 3- to 5-year patency rates range from 75% to 90% [17]. In comparison to open surgery, PTA has been documented to carry lower morbidity and mortality rate and has been established as first-line treatment of significant arterial stenosis of transplant recipients when it is viable [18].

### 4.3. Kinking

After thorough examination of the literature we have assumed that aetiology of distal stenosis is not clearly specified, yet mechanical or immunological damage seems to be related, whereas proximal stenosis is a consequence of recipient's atherosclerotic arterial disease and anastomotic stenosis is resulted from technical reasons such as “faulty surgical technique” or postoperative fibrosis [11]. In addition, kinking of the artery can occur when the artery is longer than the vein, and this may provoke or stimulate the hemodynamic and functional changes of TRAS [9, 19].

With regard to the outcomes of this paper, graft nephrectomy was avoided with the selected therapeutic approaches, and finally graft function recovery was achieved on the regular followup, indicating that PTA with stenting can accomplish similar efficacy with open surgery, when viable.

## 5. Conclusion

In summary, stenotic lesions not only in the transplanted renal artery but also more proximally or distally (close to) must be considered as source of hypertension and graft's dysfunction and should be investigated and treated with PTA when it is needed, because of the positive risk-benefit estimate of this procedure, taking into account its low invasiveness and absence of severe complications. 

The importance of immediate diagnosis and fast intervention, once the diagnosis of TRAS is established, is of major significance, while we are convinced that case-specific selection of therapeutic procedures are required for TRAS treatment. 

These cases illustrate that PTA is a reliable therapeutic approach and its high rate of clinical success even in more complicated cases establishes it as the first-line treatment in management of TRAS. Familiarity with this procedure facilitates prompt vascular diagnosis and treatment.
